# A Blind Signal Samples Detection Algorithm for Accurate Primary User Traffic Estimation

**DOI:** 10.3390/s20154136

**Published:** 2020-07-25

**Authors:** Jakub Nikonowicz, Aamir Mahmood, Mikael Gidlund

**Affiliations:** 1 Faculty of Computing and Telecommunications, Poznań University of Technology, 61-131 Poznań, Poland; 2 Department of Information Systems and Technology, Mid Sweden University, 851 70 Sundsvall, Sweden; aamir.mahmood@miun.se (A.M.); mikael.gidlund@miun.se (M.G.)

**Keywords:** discontinuous signals, blind detection, rank order filtering, primary user traffic

## Abstract

The energy detection process for enabling opportunistic spectrum access in dynamic primary user (PU) scenarios, where PU changes state from active to inactive at random time instances, requires the estimation of several parameters ranging from noise variance and signal-to-noise ratio (SNR) to instantaneous and average PU activity. A prerequisite to parameter estimation is an accurate extraction of the signal and noise samples in a received signal time frame. In this paper, we propose a low-complexity and accurate signal samples detection algorithm as compared to well-known methods, which is also blind to the PU activity distribution. The proposed algorithm is analyzed in a semi-experimental simulation setup for its accuracy and time complexity in recognizing signal and noise samples, and its use in channel occupancy estimation, under varying occupancy and SNR of the PU signal. The results confirm its suitability for acquiring the necessary information on the dynamic behavior of PU, which is otherwise assumed to be known in the literature.

## 1. Introduction

In cognitive radios, detecting white spaces, and determining channel occupancy in a dynamic radio environment is essential for opportunistic access to radio resources. The simplest and widely used method of assessing the availability of the radio resources is energy detection, for which efficiency in terms of probability of detection and probability of false alarm is analyzed extensively in the literature [1,2,3]. However, these studies consider the context of a static primary user (PU) signal, which is either active or inactive within the entire detection period. In realistic scenarios, however, the PU signal can dynamically switch between active and inactive states, while detection is in progress. The detection of discontinuous PU is considered in [4,5,6,7] by redesigning the detection algorithms, in which, however, signal, noise, and PU traffic parameters are assumed to be known a priori while the methods to obtain/estimate these necessary parameters are not described.

Many studies show that any uncertainty in estimating parameters of the received signal seriously limits the ability of the detector to assign energy to a particular activity state correctly [8]. Consequently, the correct operation of the energy detector in a dynamic radio channel requires accurately estimating, (a) PU traffic parameters such as the average and current duration of the PU, and its channel occupancy ratio and, (b) signal and noise parameters as noise variance and signal-to-noise ratio (SNR). Importantly, the estimation of all these parameters and the ensuing detection performance starts with the accurate splitting of the signal and noise samples in received energy samples.

In this paper, we present a practical algorithm for energy samples recognition—marking of signal and noise samples in a received time frame—of a dynamic PU signal. The algorithm uses rank order filtering, earlier studied for signal spectrum analysis only [9,10,11], for temporal signal analysis by redesigning the signal processing and samples marking processes. We evaluate the algorithm in terms of signal samples detection and complete samples recognition with respect to SNR and different PU activity factors, and also examine the execution time of the detection process. Besides, its performance is compared with the well-known reference methods in the literature [12,13,14], which is then followed by its utility appraisal for channel occupancy estimation. To assess the accuracy of these operations, a semi-experimental simulation setup of packet-based PU transmission is designed, where the background distortion comes from the radio frequency (RF) noise traces captured with National Instrument software defined radio (SDR), USRP-2900. The proposed solution, with its appealing performance, provides a convenient basis, although not the subject of this article, for parameter estimation in the subsequent detection of intermittent PU signals.

The rest of the article is organized as follows. Section 2 gives the motivation for samples recognition, and Section 3 describes the proposed detection algorithm. Section 4 explains the simulation methodology and shows numerical results. Finally, Section 5 gives the concluding remarks.

## 2. Motivation for Samples Recognition

**Figure 1:**
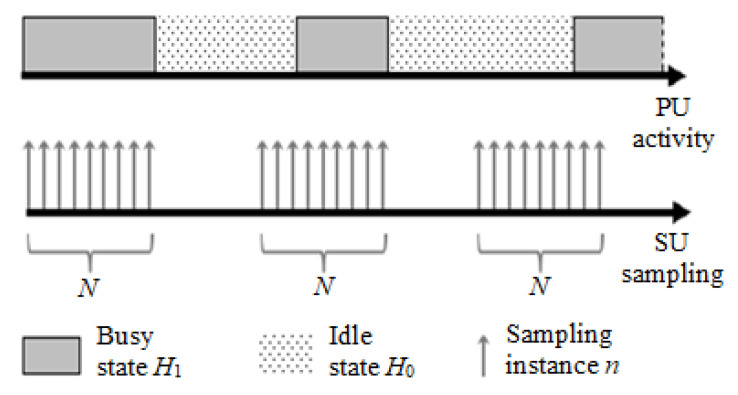
Illustration of a dynamic PU signal activity along with the energy sampling process.

To reason the need for samples recognition, we restate the energy detection (ED) process for dynamic PU signals. Consider the ED-based sampling of dynamic PU modeled as an alternating renewal process, similar to [4,5,6], and shown in Figure 1. Energy detection as an auxiliary process in basic secondary user (SU) operations is considered as discontinuous and periodic, and therefore may not register constant PU activity in the detection window, but rather may contain transitions between the activity states. At any time instant, PU is either in ON (active) or OFF (idle) state, while the state transition occurs at random time instances, and the state holding times are exponentially distributed with mean τ and μ, respectively. The energy detector collects signal samples $x_{n},n=1\cdots N$ in a detection interval of duration (*T*), which is independent of the PU ON/OFF process. As the signal is sampled at a specific frequency $f_{s}$, total number of collected samples are $N=f_{s}T$, and $N_{0}$ and $N_{1}$ represent the number of samples corresponding to hypothesis (subject to detection) $H_{0}$ and $H_{1}$. As N→∞, normalized occupancy/absence rate $N_{i}/N$ approaches its average value $p_{i},i\in \left\{0,1\right\}$.

A predominant model to characterize energy detection performance for such dynamic PU scenario, in terms of test statistic (β), probability of detection ($P_{d}$), and probability of false alarm ($P_{f}$), is as follows [4]

(1) $$\beta =p_{0}\frac{1}{N_{0}}\sum_{n=1}^{N_{0}}{\left|w_{n}\right|}^{2}+p_{1}\frac{1}{N_{1}}\sum_{n=1}^{N_{1}}{\left|s_{n}+w_{n}\right|}^{2},$$

(2) $$P_{d}=Q\left[\sqrt{N}\frac{\frac{\gamma }{\sigma_{w}^{2}}-\left(1+p_{1}\rho \right)}{\sqrt{1+p_{1}\left(\rho^{2}+2\rho \right)}}\right],$$

(3) $$P_{f}=Q\left[\sqrt{N}\left(\frac{\gamma }{\sigma_{w}^{2}}-1\right)\right],$$

where γ is the decision threshold, $\sigma_{w}^{2}$ is noise variance and ρ is the SNR, which in instantaneous form, for mixed signal-with-noise and noise-only samples, can be calculated as a ratio of averaged powers [15]

(4) $$\rho =\frac{\sum_{n=1}^{N_{1}}{\left|s_{n}+w_{n}\right|}^{2}}{\sum_{n=1}^{N_{0}}{\left|w_{n}\right|}^{2}}-1.$$

**Figure 2:**
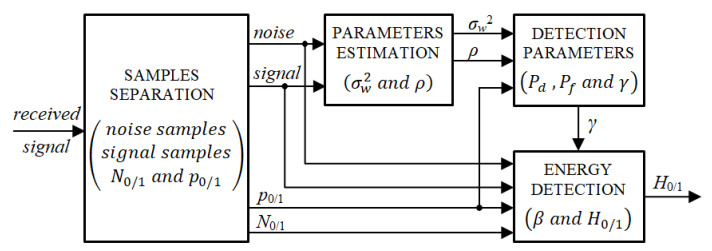
Block diagram of an energy detection process for discontinuous PU signals: source and demand of necessary parameters.

To implement this detection model or any other involving PU transition probabilities (e.g., [6]), several necessary parameters, as noise variance, SNR, instantaneous and mean occupancy/absence rates, are assumed to be known. Some of these parameters remain dependent on the primary user activity (i.e., $N_{0/1}$, $p_{0/1}$) and can be derived directly from the received signal. Others depend on the operating conditions of the receiver (i.e., $\sigma_{w}^{2}$, ρ) and must be estimated based on the appropriate groups of the received samples. In practice, the very first step to extract these parameters is samples recognition. Figure 2 summarizes the different stages of the energy detection process while featuring the source and demand of the necessary parameters at each stage.

In this context, our objective is to develop a samples separation algorithm that is effective in extracting the necessary (detection-related) parameters of bursty PU signal. We assume a specific (exponential) distribution of PU idle/active state; however, the design of the algorithm is generic and blind to the PU activity pattern.

## 3. Algorithm Design and Description

In this section, we describe the design of the proposed algorithm, which finds its motivation from rank order filtering (ROF). ROF, a commonly used image processing technique, sorts the input values in ascending order, and selects for the output value encountered at a certain rank order number. The selected input value becomes the output, without any calculation performed on the input values. The two special operations of ROF are erosion—equivalent to lowest rank as it returns the minimum of the input set, and dilation—equivalent to highest rank as it returns the maximum. Erosion and dilation, besides being useful in image processing, can also be effectively used in impulse noise reduction and noise power estimation, as demonstrated in [9,10,11]. These studies iteratively increase the size of the filters used on the power spectrum samples. By filtration, the peak values of the spectrum are reduced until the difference in the noise floor achieved in *i*–th and (i+1)–th iteration falls below a predetermined threshold value. Although effective, the algorithms in [9,10,11] are only dedicated to estimating spectrum parameters and are burdened with the following disadvantages that limit their usefulness for time-domain analysis of dynamic PU behavior:

- The selection of an appropriate threshold for the noise floor difference is problematic due to its unambiguous interpretation.
- The process carried out on spectrum samples strongly benefits from the processing gain provided by the fast Fourier transform (FFT) [16]. Although the transition between frequency and time domains can be performed quite efficiently, for simple energy detectors, it is not imperative to transform the signal into the frequency domain.

With these considerations, we design a new signal samples detecting technique, by reconstructing the ROF-based solutions, for accurate extraction of parameters from intermittent PU transmission, while keeping the samples recognition process as simple as possible to enable low-complexity detection. The main steps of the proposed algorithm are as follows:

**Initialization—energy vector**: The detection algorithm starts with the conversion of an *N* complex samples, i.e., $x=[x_{1},\cdots ,x_{N}]$, into a vector of energy samples, $y_{n}={\left|x_{n}\right|}^{2}$. Afterward, a moving average (MAV) of a small size $m_{\mathrm{init}}\ll N$ is used to reduce noise variance initially. The right choice for $m_{\mathrm{init}}$ in accordance with *N*, i.e., small enough not to reduce the signal but large enough to reduce noise variance, increases the algorithm’s accuracy for signals of longer duration or high SNR. However, too large $m_{\mathrm{init}}$ may limit the algorithm’s sensitivity for weak signals. Because the recursive formulation of the *m*-sized MAV as ${\bar{y}}_{n}={\bar{y}}_{n-1}+\frac{1}{m}\left(y_{n}-y_{n-m}\right)$ requires only one addition, one subtraction, and one division per sample, the formula is independent of the number of samples *N*, and the runtime complexity for each sample is constant, i.e., O(1). Thus, the complexity of the pre-processing preceding filtering is kept to a minimum. However, it should also be noted that the recursive implementation of the moving average can cause a risk of error propagation in the event of a calculation error, which depends on the device’s computational capabilities, i.e., limited precision. Therefore, it is a trade-off for simple devices between hardware load and the risk of introducing a faulty observation window for further processing. Nevertheless, considering the case of short observation windows, the error accumulation and propagation has a limited impact.

**LOOP process—ROF**: In this step, the initially averaged energy vector $\bar{y}$ is iteratively filtered by consecutive erosion and dilation operations. After each filtration, the total energy *e* of the vector is determined. A consistent increase in the size *k* of movmin and movmax filters allows finding the size $m_{\sec}$ for which the energy decrease in relation to the energy after previous filtration $e_{k}^{\prime }=\frac{e_{k-1}-e_{k}}{e_{k-1}}$ remains the highest. The search for maximum value removes the requirement to set a threshold, as typical in earlier works. The resulting size of the filter $m_{\sec}$ is interpreted as the probable longest continuous signal duration in the analyzed frame, and is used in the second moving average, $\bar{\bar{y}}$.

**Samples marking process—derivative evaluation**: The identification of signal and noise samples is based on the evaluation of the derivative of the double–averaged energy vector $y_{n}^{\prime }={\bar{\bar{y}}}_{n}-{\bar{\bar{y}}}_{n-1}$. The intervals in which the derivative has positive values with a width wider than the assumed threshold of minimal signal width $\lambda_{\mathrm{msw}}$ indicate signal samples. The threshold $\lambda_{\mathrm{msw}}$ can be interpreted as a resolution of the algorithm, i.e., the minimum detectable signal duration. Due to the influence of the noise variance, the problem of maintaining the required interval continuity occurs. Positive intervals potentially indicating the signal can be divided by single samples with non-positive values. Therefore, before assessing if the width of the positive interval meets the condition of $\lambda_{\mathrm{msw}}$, adjacent intervals separated by the single non-positive sample are combined. This simple step significantly improves the marking accuracy of the algorithm for weak signals.

The above-presented solution results in a simple yet effective samples recognition technique for the detection of packet-based PU signals. The pseudo-code of the proposed scheme is given in Algorithm 1 with notations: $m_{\mathrm{init}}$—initial size of the moving average, $\lambda_{\mathrm{msw}}$—minimal signal width, *x*—received signal, *y*—processed signal, *e*—energy, $e^{\prime }$—energy decrease, and $y^{\prime }$—differential.
**Algorithm 1** Pseudo-code of the samples detection algorithm**Input:***x*, $m_{\mathrm{init}}$, $\lambda_{\mathrm{msw}}$ **Output:** noise, signal  **Initialisation**:1:$y\;=\;{\left(\mathrm{abs}\left(x\right)\right)}^{2}\;$, $\bar{y}\;=\;\mathrm{mav}\left(y,m_{\mathrm{init}}\right)$, $e\;=\;\mathrm{sum}\left(\bar{y}\right)/\mathrm{length}\left(\bar{y}\right)$**Parallelized LOOP process:**2:**for**$k=2\;\mathrm{to}\mathrm{length}(\bar{y})/2$**do**3:  $erosion=\mathrm{movmin}(\bar{y},k)$4:  dilation=movmax(erosion,k)5:  $e_{k}=\mathrm{sum}\left(dilation\right)/\mathrm{length}\left(dilation\right)$6:  $e_{k}^{\prime }=\left(e_{k-1}-e_{k}\right)/e_{k-1}$7:**end for**8:$\left[value,m_{\sec}\right]=\max\left(e^{\prime }\left(2:end\right)\right)$, $\bar{\bar{y}}=\mathrm{mav}\left(\bar{y},m_{\sec}\right)$**Samples marking process:**9:**for**$j=1\;\mathrm{to}\mathrm{length}(\bar{\bar{y}})-1$**do**10:  $y_{j}^{\prime }$ = ${\bar{\bar{y}}}_{j}-{\bar{\bar{y}}}_{j+1}$11:**end for**Intervals continuity assessment:12:**for**$l=3\;\mathrm{to}\mathrm{length}(y^{\prime })$**do**13:  **if** $y_{l}^{\prime }>0$ **then**14:   $y_{l}^{\prime }=1$15:   **if** $y_{l-2}^{\prime }>0$ **then**16:     $y_{l-1}^{\prime }=1$17:   **end if**18:  **else**19:   $y_{l}^{\prime }=0$20:  **end if**21:**end for**Intervals length assessment:22:**for**$i=2\;\mathrm{to}\mathrm{length}(y^{\prime })$**do**23:  **if** $y_{i}^{\prime }$ **then**24:   $y_{i}^{\prime }=y_{i-1}^{\prime }+1$25:  **end if**26:**end for**Minimum length evaluation:27:**for**$n=1\;\mathrm{to}\mathrm{length}(y^{\prime })$**do**28:  **if** $y_{n}^{\prime }>\lambda_{\mathrm{msw}}$ **then**29:   $mark(n-\lambda_{\mathrm{msw}}:n)=1$30:  **end if**31:**end for**Samples identification:32:signal = y·mark, noise=y−signal33:**return** noise, signal

## 4. Simulation Setup and Results

To assess the performance of the proposed algorithm, we have developed a simulation setup that supports random ON/OFF traffic behavior of PU. For background noise, the simulator uses radio-frequency (RF) noise I/Q traces sampled in a bandwidth of 5 MHz centered around 868 MHz, i.e., the first channel of the IEEE 802.15.4 standard. The RF noise traces are collected in an open university area using National Instrument USRP-2900. The receiver digitizes RF samples using direct downconversion to baseband. We recorded the average noise power of −110.7 dBm, which is normalized to 1 mW in the simulations.

It should be noted that although the pseudorandom noise approach is the most commonly used in simulated transmission models, the use of pseudorandom noise, fitted to the theoretical assumptions of the white Gaussian noise, may significantly differ from the background noise recorded by the real radio receiver. The use of real noise traces includes an uneven distribution of power between frequencies, instantaneous noise power variations, and distortions due to receiver limitations.

**Figure 3:**
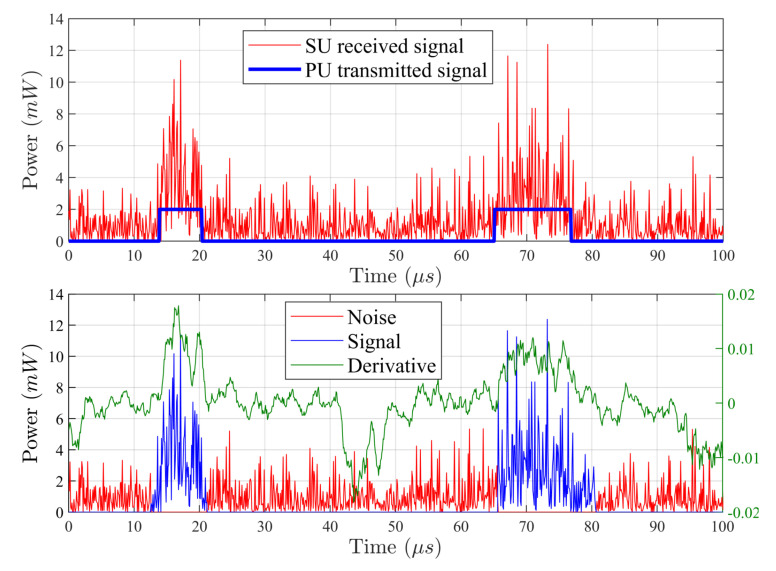
An instance of analyzed time frame: (**top**)—the time frame with RF noise and PU pulse signals with randomly distributed durations. (**bottom**)—splitting of signal and noise samples based on the processed sign of the differential.

In the simulations, the subject of analysis is non-overlapping time frames, each containing 1024 samples. A new noise realization is recorded for each frame, to which a PU signal is added as a rectangular pulse as shown in Figure 3(top). The radio pulse is adopted as a deterministic signal hidden in noise due to its universality in reproducing an unknown signal while maintaining a simple regulation of amplitude and time duration. Both the signal-with-noise pulses and the following noise-only durations are exponentially distributed with mean values being swept respectively from 10% to 30% and 90% to 70% of the time frame. The sample splitting is based on a positive or non-positive differential processed according to Algorithm 1, and depicted in Figure 3(bottom). In simulations, as input parameters of the algorithm, $m_{\mathrm{init}}$ and $\lambda_{\mathrm{msw}}$ are set as 1% and 5% of the observation window, respectively.

As the basic reference method, we study the estimation of primary user activity based on the idle/busy periods determined by using short spectrum sensing decisions [12,13,17]. As the above method, however, requires noise floor information, which we obtain using the extended Forward Consecutive Mean Excision (FCME) algorithm with Welch FFT [13,17]. For the simulation of the FCME-based algorithm, a 64-sample periodogram is adopted along with an energy detection window with a length of 5% of the analyzed time frame and a false alarm probability of 1%.

As the second reference method (operating in the time-domain), we adopt linear discriminant analysis (LDA). LDA is used in statistics and pattern recognition as a basic mathematical tool to separate two classes of objects, applied as Fisher discriminant function [14].

**Figure 4:**
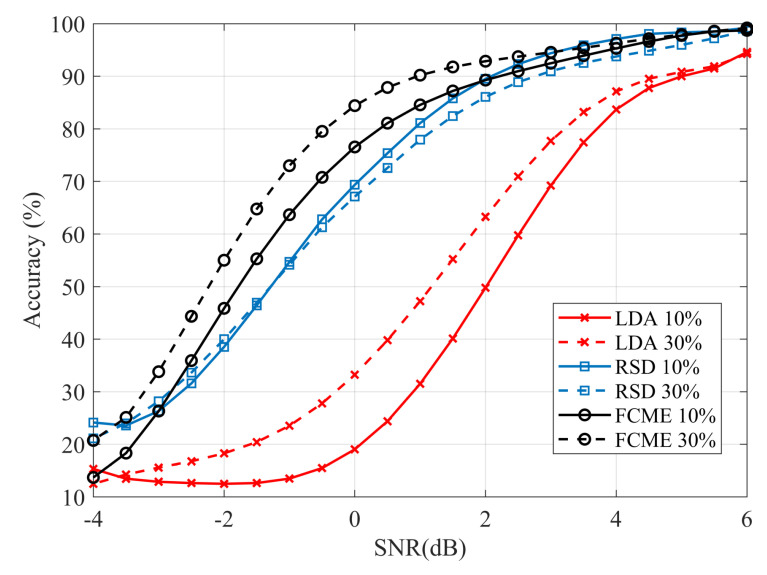
Signal samples detection efficiency as an average ratio of correctly recognized signal samples.

**Figure 5:**
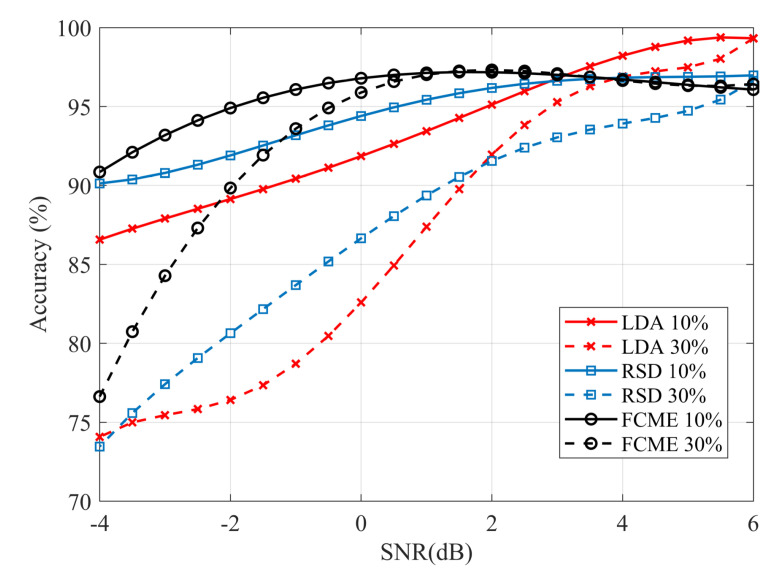
Total detection efficiency as an average ratio of correctly recognized signal-with-noise and noise-only samples.

The performance of the algorithms is measured by the average percentage of correctness in assigning samples to groups of signal-with-noise or noise-only samples, compared to a known pattern generated independently for each time frame. Each comparison point in Figure 4 and Figure 5 is obtained after averaging 1000 observations, generated according to point parameters. In the simulated SNR dependencies, the variability of the $N_{0}$ and $N_{1}$ ratio in Equation (4) should be considered. Therefore, the SNR value is determined as the average value obtained for all observations at the assumed constant pulse amplitude set relative to the average noise amplitude, and taking into account the average duration of the pulse generated with a given τ.

Figure 4 shows the detection accuracy for the signal group, indicating a decrease in the assignment accuracy with a decrease in SNR. The adopted metric approximates the probability of detection as N→∞. The comparison indicates that the proposed ROF-based signal detection (RSD) shows a performance near to FCME-based solution and significantly higher than LDA (i.e., the reference time-domain method). Moreover, the accuracy of RSD remains almost independent of the mean occupancy of the time frame.

The total marking accuracy, measured as an average ratio of correctly recognized signal-with-noise samples and noise-only samples, is shown in Figure 5. With a decrease in SNR, the curve indicates an increase in type I and type II errors. The efficiency of RSD decreases by the percentage close to the signal presence in the frame, which in extreme cases is entirely recognized as noise. Thus, the inaccuracy in RSD is mainly limited to error type false negative, i.e., less recognizable signal samples are assigned to the noise group, while the noise is rarely classified as a signal, leading to reduced false positive errors. At high SNR, the accuracy does not drop more than the assumed 5% resolution threshold.

**Figure 6:**
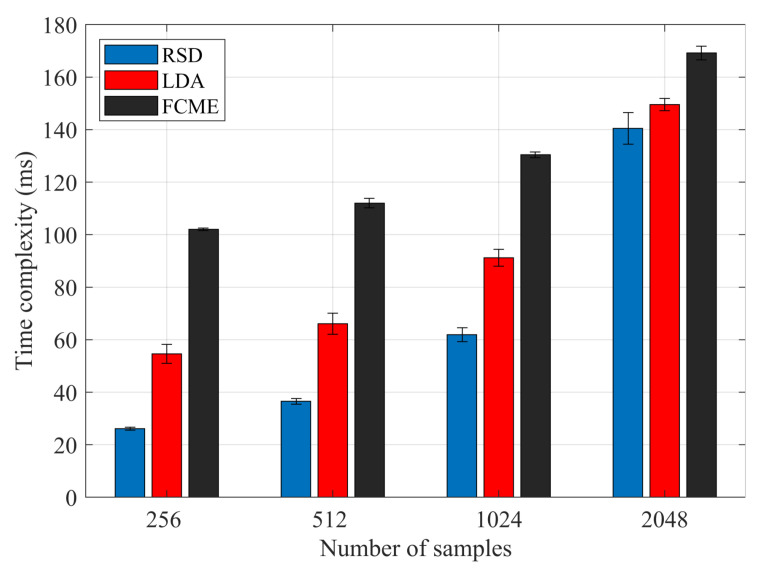
Time complexity as an average execution time of the detection process for a given number of samples.

To complement the comparison, it is also necessary to analyze the complexity of the methods. However, for short observation windows, thus small number of samples, such as those used in this study, the asymptotic behavior of the methods may not dominate. The differences in performance can be caused by time-consuming operations other than calculations, i.e., bringing the data into and out of the processor cache. Therefore, the use of asymptotic analysis for practical *N* values has limited utility. Accordingly, Figure 6 compares the average execution time of the proposed RSD algorithm with the reference methods. The results were obtained by averaging the time over a thousand single-threaded function calls performed on a quad-core 3.07 GHz Intel Xeon W3550. Time analysis shows that the proposed RSD method exhibits significantly lower complexity, especially for a small number of samples, with respect to the reference solutions. These time differences are of significant importance as the samples recognition remains only a supportive process for effective estimation of channel parameters and primary user detection.

**Figure 7:**
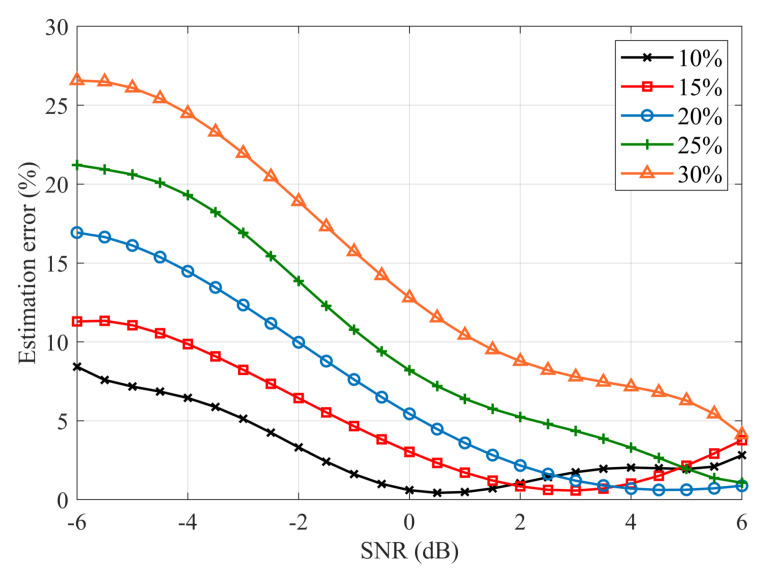
Accuracy of the mean channel occupancy estimation.

The marking efficiency of signal-with-noise samples in the exemplary application can be directly used for estimating the average occupancy of the PU signal under test. For each observation window, based on the algorithm’s indications, the number $N_{1}$ of signal-with-noise samples is determined. Samples are collected over 1000 observation windows and based on them, the average occupancy of the transmission channel is determined. Then the relative error of the estimated channel occupancy is determined relative to the expected value τ for which the frames were generated. As shown in Figure 7, it is visible that for a low PU occupancy of 10–20% with SNR above 0 dB, the proposed algorithm preserves high 1–2% accuracy. In the case of increasing occupancy and weak signals, the algorithm noticeably loses efficiency. Therefore, for a secondary user subject to strong PU signals with a moderate activity factor, the proposed algorithm demonstrates to be a perfectly matched, easy to implement, and accurate signal samples detection technique.

## 5. Conclusions and Future Work

Stimulated by the need for estimating several vital parameters to perform the energy detection of dynamic PU, we developed a new algorithm for accurate recognition of signal and noise samples in the received signal time frame. The algorithm has its roots in rank order filtering-based spectral analysis that we reconstructed for low-complexity time-domain analysis of bursty signals. We evaluated the algorithm in terms of its accuracy to detect signal samples, complete sample recognition, time complexity and utility in channel occupancy estimation. The algorithm exhibits an accuracy of 87% in marking samples even for weak signals with SNR close to 0 dB. For strong and narrow pulses, it provides up to 97% of correct sample recognition and remains competitive for twice as complex solutions. The achieved accuracy, together with a simple design, makes the proposed solution a convenient basis for obtaining information required for effective energy detection.

Future fully real-data experiments can provide detailed information related to the impact of the remaining operational parameters, i.e., $m_{init}$ and $\lambda_{msw}$ and their optimization to enhance the overall accuracy of the proposed solution. As a future work, we also aim to consider variable sensing periods and transmission power levels, and multiple PUs.

## Abbreviations

The following abbreviations are used in this manuscript:

ED	energy detection
FCME	Forward Consecutive Mean Excision
FFT	fast Fourier transform
LDA	linear discriminant analysis
MAV	moving average
PU	primary user
RF	radio frequency
ROF	rank order filter
RSD	ROF-based signal detection
SDR	software defined radio
SNR	signal to noise ratio
SU	secondary user

## References

[1] Urkowitz H. (1967). Energy detection of unknown deterministic signals. Proc. IEEE.

[2] Sharma S., Bogale T., Chatzinotas S., Ottersten B., Le L., Wang X. (2015). Advances on spectrum sensing for cognitive radio networks: Theory and applications. IEEE Commun. Surveys Tutor..

[3] Ali A., Hamouda W. (2017). Advances on spectrum sensing for cognitive radio networks: Theory and applications. IEEE Commun. Surv. Tutor..

[4] Penna F., Garello R. (2011). Detection of discontinuous signals for cognitive radio applications. IET Commun..

[5] Saad W., Ismail M., Nordin R., El-Saleh A. (2016). Spectrum sensing schemes for dynamic primary user signal under AWGN and Rayleigh fading channels. J. Commun..

[6] MacDonald S., Popescu D., Popescu O. (2017). Analyzing the performance of spectrum sensing in cognitive radio systems with dynamic PU activity. IEEE Commun. Lett..

[7] Duzenli T., Akay O. (2019). A new method of spectrum sensing in cognitive radio for dynamic and randomly modelled primary users. IETE J. Res..

[8] Mariani A., Giorgetti A., Chiani M. (2011). Effects of noise power estimation on energy detection for cognitive radio applications. IEEE Trans. Commun..

[9] Taher T., Attard R., Riaz A., Roberson D., Taylor J., Zdunek K., Hallio J., Ekman R., Paavola J., Suutala J. Global Spectrum Observatory Network Setup and Initial Findings. Proceedings of the 2014 9th International Conference on Cognitive Radio Oriented Wireless Networks and Communications (CROWNCOM).

[10] Agarwal A., Sengar A., Debnath S. A novel noise floor estimation technique for optimized thresholding in spectrum sensing. Proceedings of the 2017 International Conference on Advances in Computing, Communications and Informatics (ICACCI).

[11] Nikonowicz J., Mahmood A., Sisinni E., Gidlund M. (2019). Noise Power Estimators in ISM Radio Environments: Performance Comparison and Enhancement Using a Novel Samples Separation Technique. IEEE Trans. Instrum. Meas..

[12] Toma O., Lopez-Benitez M., Patel D., Umebayashi K. (2020). Estimation of Primary Channel Activity Statistics in Cognitive Radio Based on Imperfect Spectrum Sensing. IEEE Trans. Commun..

[13] Iwata H., Umebayashi K., Al-Tahmeesschi A., Lopez-Benitez M., Lehtomaki J. Time and Frequency Varying Noise Floor Estimation for Spectrum Usage Measurement. Proceedings of the 2019 IEEE Wireless Communications and Networking Conference Workshop (WCNCW).

[14] Chien J.T. (2019). Source Separation and Machine Learning.

[15] Huszty C., Sakamoto S. (2012). A note on the definition of signal-to-noise ratio of room impulse responses. Acoust. Sci. Technol..

[16] Lyons R.G. (2004). Understanding Digital Signal Processing.

[17] Umebayashi K., Takagi R., Ioroi N., Suzuki Y., Lehtomaki J. Automatic noise floor spectrum estimation in the presence of signals. Proceedings of the Conference Record of the Thirty-First Asilomar Conference on Signals, Systems and Computers (Cat. No.97CB36136).

